# Review on Hypothetical Implementing TGF-β Family Members in Glaucoma Therapy

**Published:** 2012

**Authors:** Ivan Sosa, Kata Culina, Alan Bosnar

**Affiliations:** 1 Department of Forensic Medicine and Criminalistics; Rijeka University School of Medicine; Rijeka; Croatia; 2 Ophthalmologist at “Okulisticki Centar”; Zagreb; Croatia

**Keywords:** Endothelium, Neuroprotection, Ocular Blood Flow, Transforming Growth Factor – β, Vascular Theory on Glaucoma

## Abstract

For quite some time, glaucoma has been regarded as more than just intraocular pressure [IOP] elevation. Significant contribution to this conceptual improvement has risen from a better understanding of ocular blood flow, vessel wall integrity and certain advanced ideas in neuroophthalmology, for example neuroprotection. Transforming growth factor–β (TGF-β) molecule, its inhibitors and antagonists have been increasingly researched as possible new anti-glaucoma drugs for its many, pleiotropic, effects. Among those effects, enhancing fibrosis is one of the most apparent, but certain members of this cytokine’s superfamily act as anti-fibrotics. Recent scientific efforts strongly support pushing back the frontier of conventional medical treatment. Current medical approaches already use effects on blood flow and neuronal quiescence, with significant systemic side-effects. Endeavours on the ophthalmologic exploitation of selected, favourable effects of pleiotropic TGF-βs could promote TGF-β, its inhibitors or specific antibodies as new, ideal drugs in glaucoma therapy.

## INTRODUCTION

Glaucoma, a progressive optic neuropathy [1,2], is the second leading cause of vision loss. The vascular theory of glaucoma considers optic neuropathy as a consequence of blood supply that is jeopardised by a reduced ocular blood flow [3,4]. Ocular blood flow is an extremely complex process, as metabolic needs follow changes in visual function [5-10]. 

In vitro studies have suggested that transforming growth factor-β [TGF-β] signalling pathways regulate angiogenesis [11,12]. This originates from ALK-1 [13] and -5. Both act through receptor-regulated SMADs, though via different methods [14]. Mostly, SMADs are bone morphogenetic protein [BMP]-dependent, and are activated in various animal tissues [15]. In pulmonary or hepatic fibrosis, systemic sclerosis, glomerulosclerosis or in dermal scarring, there is noticeable evidence that TGF-β mediates a pathological increase in extracellular matrix deposition [16-20]. Although not all members of this superfamily act as pro-fibrotics [12,15,16,20], TGF-β was found to increase extracellular matrix proteins in the optic nerve [21], and affect rabbit sub-conjunctival fibroblasts [22]. 

**Endothelium**

BMPs play an important role in endothelial cell [EC] function [23-26]. Interestingly, different ECs are differently susceptible to different isoforms of TGF-β. BMP-4 and -6 [members of the TGF-β superfamily] promote EC migration and proliferation [27], while BMP-9 is a circulating vascular quiescence factor [28]. Vascular endothelium releases different vasoactive factors that regulate the microcirculation [29,30]. Previously, BMP-2, -4, and -7 have been reported to bind ALK1 receptors and EC, which are targets for certain ligands of the BMP members of the TGF family [23]. Vascular endothelial dysfunction is a frequent basis of many diseases [31,32]. Dysfunction in the endothelium can influence the vessel’s diameter and resistance. 

Reduced levels of nitric oxide [NO] can result in decreased vasodilatation and increased vasoconstriction [33-35] connected consecutively by a decrease in nitrosylation [36] and fragmentation of DNA, all of which lead to apoptosis [37]. Low levels of nitric oxide reduce blood flow as in glaucoma. Compromised availability of NO as well as an imbalance between NO and endothelin-1 [ET-1] have been reported in glaucoma patients [38]. Patients with normal-tension glaucoma have increased plasma, and those with open angle glaucoma have aqueous humor levels of ET-1 [39,40]. Vasoconstriction inevitably leads to hypoxia, which makes it reasonable to suppose that various cytokines may be up-regulated in glaucoma [41,42].

**Ocular Blood Flow**

Researchers have long reported that patients with open-angle glaucoma exert altered blood flow in retinal, choroid, and retro-bulbar circulation [5-9,43]. An alteration in the eye blood supply can be further correlated to vascular endothelial dysfunction [31,32]. The narrowing of blood vessels increases resistance to flow distally, which leads to hypoxia. Several population-based studies documented retinal vascular narrowing. Structural changes might increase flow resistance, or might result in functional dysregulation of the vascular width. Reduction in the blood flow is not only limited to the eye but to the orbit and even the periphery. In some patients, blood flow reduction precedes glaucoma [44]. Intraocular pressure [IOP] alone is unlikely to cause the disruption of ocular blood flow more distinctly in normal-tension patients than high-tension ones. Reduced perfusion pressure could result in increased IOP or decreased blood pressure [44-46], and the increased viscosity of blood can be a result of a blood dyscrasia.

**Neuroprotection of TGF-β**

Due to its pleiotropy, the beneficial effect of TGF-β on vascular integrity has been easy to understand. That effect is not impossible to link to its many different functions, like local neuroprotective humoral agents or mediator in embryogenesis. The objective is to connect its vascular quiescence to the established endothelial NO production in order to influence cerebral perfusion [48]. Furthermore, TGF-β as the vascular-integrity guard ensures the preservation of the vessel wall, thus eliminating factors required for scarring. TGF-β mRNA is elevated for at least a week after a stroke and clearly exerts a neuroprotective role [48]. Nevertheless, in order to exploit the therapeutic properties of TGF-β, any additional roles in the brain after stroke should be clearly understood. However, the acute abolition of blood supply, as in strokes, should not be compared to long-lasting, chronic diseases, like glaucoma. Moreover, not all TGF-β members exhibit pro-fibrotic actions. 

Thanks to the neuroprotective effects of TGF-β [47-49], it is considered a future important target for therapy following a stroke. The precise function of increased TGF-β after stroke is unknown and due to its pleiotropic nature, it might well modulate NO production by ECs, orchestrate glial scarring, or function as a significant immune system regulator. Even NO is potentially neurotoxic, due to the reaction with superoxide anions, which produce reactive free radical species.

## HYPOTHESIS

The ideal anti-glaucoma drug would prevent cell death of the retina with no adverse effects. However, a more realistic ideal drug would be one that reduces IOP, and reaches the retina in appropriate amounts to reduce retinal cell death, even if applied topically [2,45,50]. A variety of agents may act on growth factors, including TGF-β. Employing antibodies against the TGF-β superfamily members that are involved in angiogenesis and vessel quiescence as a treatment option for glaucoma is not new. One of the theoretical advantages of the human TGF-β2 antibody [which is the predominant form in the aqueous] is that it only acts if there is TGF-β2 in the wound, unlike an anti-metabolite [51]. Even if all of the above is neglected, the vasoconstrictive effect of ET-1, mediated by TGF-β-stimulated ALK-5 remains an open option. The employment of inhibitors of TGF-β in glaucoma therapy is also not new. N-[3', 4'—dimethoxycinnamoyl]anthranilic acid inhibits TGF-β activity and has anti-scarring effects in the body and the eye [52]. Interferon-α, an anti-fibrotic cytokine, has been shown to reduce the scarring activity of fibroblasts, although a clinical trial did not show it to be significantly better than current anti-metabolites [53].

With respect to vascular theory on the aetiology of glaucoma, the possible involvement of the TGF-β signalling system in the treatment approach of glaucoma is hypothesised, regarding endothelial dysfunction, ocular blood flow and neuroprotective features of TGF-β. This should be based on the inhibition of the pathological accumulation of the extracellular matrix and the modulation of fibrotic mechanisms with new anti-fibrotic agents. Further confirmation is needed to elucidate whether the cross-talk of TGF-β with other pathway systems already employed in the therapy of glaucoma exists. Also, it is important to fully understand any possible impact of any TGF-β superfamily members on complex pathophysiological mechanisms in glaucoma. 

## DISCUSSION

The vascular theory suggests that insufficient blood supply results in glaucoma. Conditions such as normal-tension glaucoma are among the chief strongholds to this theory. The reduction of ocular blood flow often precedes glaucomatous damage. Other parts of the body in glaucoma patients might also exert reduced blood flow, e.g. the extremities. Additionally, there is an increased prevalence of ischaemic lesions in other organs of the body that result in hearing problems, heart attacks, and small strokes in these patients.

Vascular dysregulation in arteriosclerosis leads to low profusion. Glaucoma is only faintly related to arteriosclerosis. The relationship between treatment with antihypertensive medication in glaucoma-free subjects and structural changes in the optic disc was established by epidemiologic cross-sectional studies [44,54]. Increased IOP is, to some extent, associated with high blood pressure, but glaucoma is linked to low blood pressure [44]. Finally, patients with a decrease in blood pressure while sleeping may have a higher risk of glaucoma progression. Low perfusion pressure is compensated by auto-regulation, ensuring normal perfusion. There are indications that this auto-regulation is altered in some patients with glaucoma. 

Vascular conditions, such as Raynaud’s disease, apnoea during sleep [55], and migraine headaches, are all associated with glaucoma. Raynaud’s disease may be an indicator for normal-tension glaucoma. Sleep apnoea is not necessarily a vascular condition, although decreased breathing means diminutions in the intake of oxygen. Migraine headaches may indicate decreased blood flow to areas of the brain. Atrial fibrillation, possibly causing irregular flow states, is also associated. Loss of blood, haemorrhage, or the necessity for a blood transfusion, can also cause glaucomatous defects in the optic nerve and visual field [56,57]. 

It is almost certain that obesity would be risk factor for both increased IOP and for arteriosclerosis. High cholesterol is also a risk factor for increased IOP, but not for normal-tension glaucoma. Normal-tension glaucoma patients tend to have a lower body mass index [58], and smoking and diabetes are associated with increased IOP but not with glaucoma. Men tend to have more, and earlier, arteriosclerotic plaques, but women are actually at a higher risk for normal-tension glaucoma [59,60]. 

Indirect signs of altered blood flow in the eye include changes in conjunctival capillaries. Something less evident, however, is local vasoconstriction in the retina. An increased frequency of optic disc haemorrhages and gliosis-like alterations are indicators for altered blood flow. Disc haemorrhages occur in all stages of the disease and are more frequent in normal-tension glaucoma [61]. 

Since the heart cycle significantly reflects the arterial blood flow to the eye, the volume of all ocular sheets [especially of the choroid] and the IOP are highest during systole [62]. A connection of vasculature and glaucoma should be sought in a decrease of blood flow [5-9,31,32,43]. Decreased blood influx to the optic nerve might be either due to decreased blood pressure, narrowing of the vessels or due to increased IOP [41,44]. Decreased circulation follows decreased blood flow or decreased perfusion, which could result in increased optic nerve damage that is comparable to ischaemia of the heart or brain [30,41].

Medications could be used to lower IOP. Several different classes of medications are used in glaucoma management [63,64]. None of these is unfettered with both local and systemic side-effects. If they occur, the patient must be willing either to tolerate these, or to communicate with the treating physician to improve the drug regimen. Poor compliance with treatment regimes and follow-up visits is a major reason for disease progression to blindness in glaucoma patients. Patient education and communication must be the target of any successful therapy.

Various cytokines are released as a response to tissue injury [65], i.e. a breakdown in the blood—aqueous barrier [66], which may not be clinically visible. This feature was established for numerous agents active on a cellular level, including cytokines and growth factors. Because of their significance, tissue scarring, inhibition or antagonism of this action was the focus of glaucoma therapy. 

Therefore, in the therapy of glaucoma, fibrinolytic agents have had their place [67] as they may lower IOP, however, a risk of further extra- and intraocular haemorrhage should not be neglected [68]. Fibroblast activation has also been a target of anti-glaucoma therapy [69].

However, considering all of these molecules, they may have a longer-term stimulatory effect on wound healing when breaking down. The prevention of clotting may be a more promising avenue.

Gingko biloba extract is one of the supplements that has been given to patients most frequently [70,71], and may increase peripheral blood flow [72]. A diet high in antioxidants has been believed to reduce the risk of glaucoma [73]. However, none of the supplements have been proven in randomised controlled trials. 

It is possible that, irrespective of IOP, various strains can damage the connective tissue of the optic nerve and axons. Reduced ocular blood flow, perhaps adjoined by underlying vascular disease, is directly involved in the pathophysiology of glaucoma. Dysregulation or an inadequate vasomotor activity in some glaucoma patients is often combined with widening vessels in neighbouring tissues. This decreases the adjusting capacity for the different flow states in decreasing blood flow to the nerve, and thereby causes the subsequent progression of the glaucoma. 

Certain carbonic anhydrase inhibitors have been shown to possibly increase ocular perfusion and produce a short-term improvement in the visual field [56,63]. The potential neuroprotective effects of various topical and systemic medications are also being investigated. In this way, the role of TGF-β, its inhibition or deleting its effect by coupling with polyclonal antibodies, needs to be viewed and evaluated as a possible ideal drug for glaucoma therapy.

## CONCLUSIONS

There is a need to improve the technology for the measurement of ocular blood flow, since studies are still at the stage of simply lowering IOP [45]. Next, a requirement exists for establishing data by comparing glaucoma patients to normal individuals and to identify subgroups of those with glaucoma. After setting out all of the parameters, it will be possible to correlate these findings with optic nerve and visual field damage. That might, perhaps target vascular dysregulation to treat glaucoma patients. At present, no medication exists to increase ocular perfusion. 

Neuroprotection is appreciated for the very absence of the need to treat the cause of the disease. Neuroprotective medications are, however, still being explored, and could provide protection to such neurons that continue to remain at risk. Neuroprotection attempts to address the common way of an insult, regardless of its cause.

The neuroprotective role of α2-adrenergic receptor agonists in the retina seems to be gaining importance in the treatment of glaucoma. However, the exact mechanism [supposedly transactivation] by which an α2-adrenergic agonist exhibits neuroprotection of neuronal elements in the retina still remains to be proven [74].

Endothelial integrity, blood flow, and glaucoma are active and dynamic fields that will, in the future, given adequate attention, interest, and scientific research, produce results that will help treat and withhold the progression of glaucoma. 

## DISCLOSURE

The authors report no conflicts of interest in this work.
